# Mechanism of substrate recognition by the novel Botulinum Neurotoxin subtype F5

**DOI:** 10.1038/srep19875

**Published:** 2016-01-22

**Authors:** Jiubiao Guo, Edward Wai Chi Chan, Sheng Chen

**Affiliations:** 1 Shenzhen Key lab for Food Biological Safety Control, Food Safety and Technology Research Center, Hong Kong PolyU Shen Zhen Research Institute, Shenzhen, P. R. China; 2 State Key Lab of Chirosciences, Department of Applied Biology and Chemical Technology, The Hong Kong Polytechnic University, Hung Hom, Kowloon, Hong Kong

## Abstract

Botulinum Neurotoxins (BoNTs) are the causative agents of botulism, which act by potently inhibiting the neurotransmitter release in motor neurons. Seven serotypes of BoNTs designated as BoNT/A-G have been identified. Recently, two novel types of Botulinum neurotoxins, which cleave a novel scissile bond, L^54^-E^55^, of VAMP-2 have been reported including BoNT/F subtype F5 and serotype H. However, little has been known on how these BoNTs recognize their substrates. The present study addressed for the first time the unique substrate recognition mechanism of LC/F5. Our data indicated that the optimal peptide required for efficient LC/F5 substrate cleavage is VAMP-2 (20–65). Interestingly, the overall mode of substrate recognition adopted by LC/F5 was similar to LC/F1, except that its recognition sites were shifted one helix toward the N-terminus of VAMP-2 when compared to that of LC/F1. The composition of LC/F5 pockets were found to have changed accordingly to facilitate specific recognition of these new sites of VAMP-2, including the P2′, P1′, P2, P3, B3, B2 and B1 sites. The study provides direct evidence of the evolutionary adaption of BoNT to recognize its substrate which is useful for effective antitoxin and inhibitor development.

Botulism, named after the Latin word “*botulus*” for sausage, was first described by Justinus Kerner after a food poisoning outbreak associated with ingestion of blood sausages^1^. Symptoms of botulism, which often originated from food or wound infection, are attributed to the most potent protein neurotoxin known to mankind, namely Botulinum Neurotoxins (BoNTs), which are produced by the bacterium *Clostridia botulinum*^2^. Human botulism is serious and fatal. If prompt diagnosis and immediate treatment are not given, the mortality rate of botulism can reach 5–10%; hence early detection and diagnosis is the key to preventing botulism. Moreover, due to its stability and easy-to-deliver/synthesize characteristics, BoNTs are regarded as potential biological weapons in bioterrorism attacks and listed as Category A agent by the Center for Disease Control and Prevention in the United States^3^,^4^.

BoNTs are typical neurotransmitter release blocker which inhibits the release of acetylcholine (Ach) by specifically targeting and degrading the SNARE (Soluble N-ethylmaleimide sensitive fusion protein (NSF) Attachment protein Receptor) proteins at the neuromuscular junction. BoNTs belong to the AB toxin family, in which the holotoxin is a ~150 kDa single polypeptide chain which can be functionally divided into two domains: an N-terminal ~50 kDa light chain (LC, catalytic domain) and a ~100 kDa C-terminal heavy chain (HC), both of which are covalently linked through a disulfide bond until they encounter reducing conditions in the neuronal cytosol^5^. The HC domain is composed of two sub-domains: translocation domain H_N_ which mediates translocation of LC across the endosomal membrane, and cell surface receptor-binding domain H_C_^6^,^7^. In the past decade, seven serotypes of BoNTs (designated as BoNT/A-G) and 31 BoNT subtypes/variants have been identified^2^,^8^,^9^,^10^,^11^,^12^,^13^,^14^,^15^,^16^. In addition, an eighth serotype, BoNT/H, has been reported^17^ and subsequently confirmed to be a hybrid of serotypes F and A characterized with BoNT/A antigenicity and BoNT/F5 light chain function^18^.

Recently, two novel BoNTs, BoNT/F subtype F5 and serotype H, which cleave a novel scissile bond of VAMP-2 (Vesicle-associated membrane protein 2), namely L^54^-E^55^, have been identified. The VAMP-2 is highly conserved and brain-specific, play key role in neuron exocytosis by forming the core SNARE complex with some other proteins^19^,^20^. BoNT/F5 exhibits highest homology to serotype F with 46–49% identity in amino acid sequence to the other six subtypes within the BoNT/F serotype^21^,^22^. It cleaves the substrate VAMP-2 at a different scissile bond from all the other six subtypes, suggesting that it may belong to a novel class of BoNT in terms of biochemical activity. BoNT/H has recently been reported by California Department of Public Health. The novel toxin gene contains regions of similarity to both *bont* /A1and *bont* /F5. The LC domain of BoNT/H is 83% homologous to LC/F5, while the HCc domain is nearly identical (>90%) to *bont/* A1. However, the HC_N_ domain of this hybrid is less similar (ie <80%) to the HC_N_ of either *bont/*A1 or *bont*/F5, which is different from other hybrid BoNT such as C/D hybrid^23^. Consistently, the toxic effects of this hybrid-like BoNT are completely eliminated by existing serotype A antitoxin. However, little has been known on the substrate recognition mechanisms of this novel toxin. In the present study, we investigated the substrate recognition and cleavage mechanism of one of this novel toxin, LC/F5. Findings in the present work could broaden our understanding of the substrate recognition mechanism utilized by BoNTs and provide insights into the development of inhibitors of the novel BoNTs.

## Results

**Figure 1:**
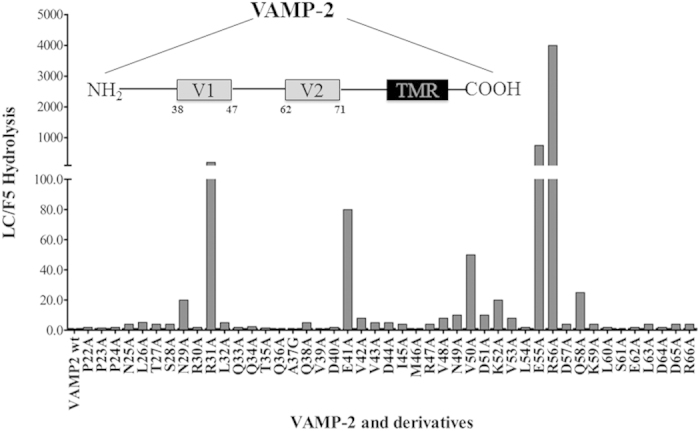
Analysis of efficiency of LC/F5 cleavage of VAMP-2 and derivatives. Hydrolysis rate was measured as the ratio of the amount of LC/F5 to cleave 50% of VAMP-2 derivatives/VAMP-2 wt. In the inlet, the motifs distribution within VAMP-2 is illustrated.

Since LC/H showed over 80% homology with LC/F5, we selected LC/F5 to study the mechanisms of substrate recognition for these novel BoNTs. GST tagged LC/F5, namely GST-LC/F5 (1–450), was shown to be the most functionally active recombinant protein^24^, it is used for this study. First, the minimal substrate for LC/F5 was determined through screening for the truncation mutations of VAMP-2. VAMP-2 (20–97) and VAMP-2 (1–65) were as efficient as WT VAMP-2 to be cleaved by LC/F5, therefore the minimal substrate was determined to be VAMP-2 (20–65). Next, saturation mutagenesis analysis of VAMP-2(22–66) was performed to dissect the role of individual residue on LC/F5 substrate recognition (Fig. 1). Several residues within the minimal substrate was shown to be critical for LC/F5 substrate cleavage including R^31^, E^41^, N^49^, V^50^, D^51^, K^52^, E^55^, R^56^ and Q^58^, substitution of which to Ala caused ~200-, 80-, 10-, 50-, 10-, 20-, 750-, 4000- and 25-fold reduction of LC/F5 substrate cleavage respectively (Fig. 1), with most of these residues being located within or adjacent to the V1 motif of VAMP-2 (Fig. 1, inlet).

**Figure 2:**
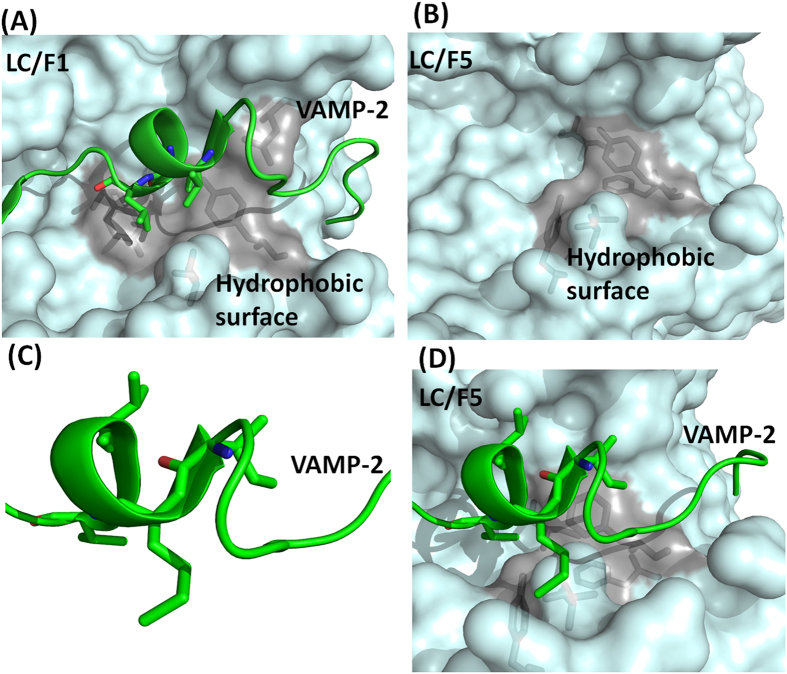
The predicted hydrophobic interactions between the α-helix of VAMP-2 and LC/F1 and LC/F5. (**A**) The LC/F1-VAMP-2 complex structure indicates that a helical structure in VAMP-2 interacts with LC/F1 through a hydrophobic interface. (**B**) The modeled structure of LC/F5 is established by using the structure of LC/F (chain A, PDB 2A8A) as search model, with the predicted hydrophobic surface illustrated. (**C**) A shifted helical structure comprised of hydrophobic residues is observed in VAMP-2 based on the modeled LC/F5-VAMP-2 complex structure. (**D**) The merged LC/F5-VAMP-2 complex structure from B and C, showing the hydrophobic interactions between the shifted α-helix of VAMP-2 and the corresponding hydrophobic pocket in LC/F5. VAMP-2 is shown in cartoon, with the hydrophobic residues in the α-helix of VAMP-2 being shown in sticks. The LC/F1 and LC/F5 are shown on the surface, with the hydrophobic residues that interact with the corresponding residues in the α-helix of VAMP-2 shown in sticks and surface in gray.

**Figure 3:**
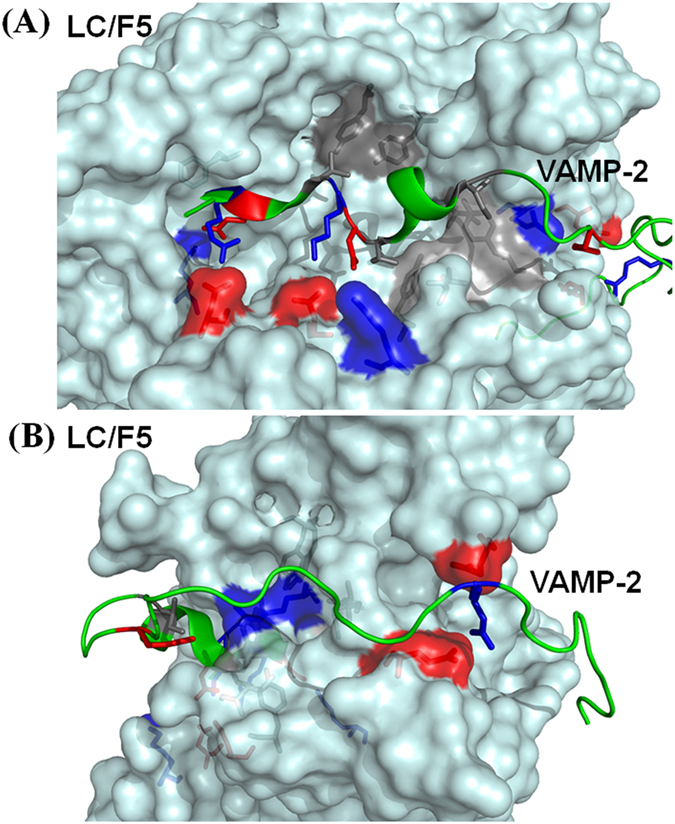
Overall view of the modeled LC/F5-VAMP-2 complex structure. (**A**) view of the active site, B2 and B3 region/site alignment; (**B**) view after a 90° clockwise turn, displaying the B1 site interactions. LC/F5 is shown as a surface structure, and VAMP-2 in cartoon. Based on prediction, the corresponding interacting sites/regions between LC/F5 and VAMP-2 are highlighted. The residues were colored based on the nature of side chain: negatively charged (red), positively charged (blue) and hydrophobic (gray).

**Figure 4:**
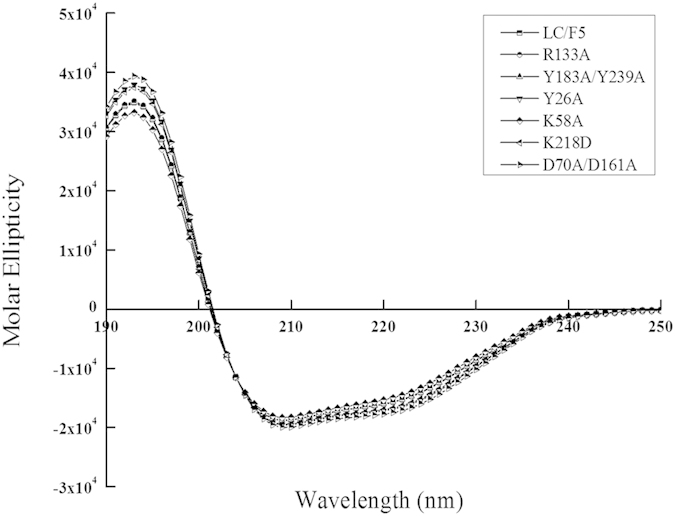
Circular Dichroism spectroscopy analysis of LC/F5 derivatives. Far-UV CD (190–250 nm) data were obtained for LC/F5 and its derivatives with a JASCO J-810 spectropolarimeter at room temperature. The molar ellipticity per residue weight, each of which is labeled with different symbol, is shown for the representative LC/F5 derivatives and wild type.

To understand the mechanism of LC/F5 substrate recognition and specificity, the complex structure of LC/F5 and VAMP-2 needs to be determined. However, the three-dimensional complex structure of LC/F5-VAMP-2 is not available currently. Instead, the LC/F5-VAMP-2 complex structure was modeled in the present work. LC/F5 and LC/F1 display a high degree similarity (~48%) in sequence, and the detailed interactions between LC/F1 and VAMP-2 have been dissected previously which was featured by a hydrophobic interaction between a helical structure of VAMP-2 and a hydrophobic interface in LC/F1 (Fig. 2A)^25^. By utilizing these information and findings, the LC/F5 structure (Fig. 2B) and the LC/F5-VAMP-2 complex structure (Fig. 2D) were modeled. As expected, a hydrophobic surface and an N-terminus shifted helix structure were identified in LC/F5 (Fig. 2B) and VAMP-2 (Fig. 2C) respectively. Based on the modeled LC/F5-VAMP-2 complex structure, the residues that may specifically interact with the corresponding residues in VAMP-2 were identified and characterized (Fig. 3) to confirm their functional roles through mutational analysis. All LC/F5 mutants were found to exhibit a similar secondary structure profile as that of the wild type LC/F5 protein through far-UV CD analysis (Fig. 4) and partial trypsin digestion (data not shown), suggesting that the conformation of the mutant proteins remains stable.

### LC/F5 active site substrate recognition

Based on the modeled complex structure of LC/F5-VAMP-2, four potential substrate recognition pockets, S2′, S1′, S2 and S3 that specifically interact with P2′, P1′, P2 and P3 sites of VAMP-2 were identified and further characterized (Fig. 3).

#### LC/F5 S2, -P2, substrate recognition

**Figure 5:**
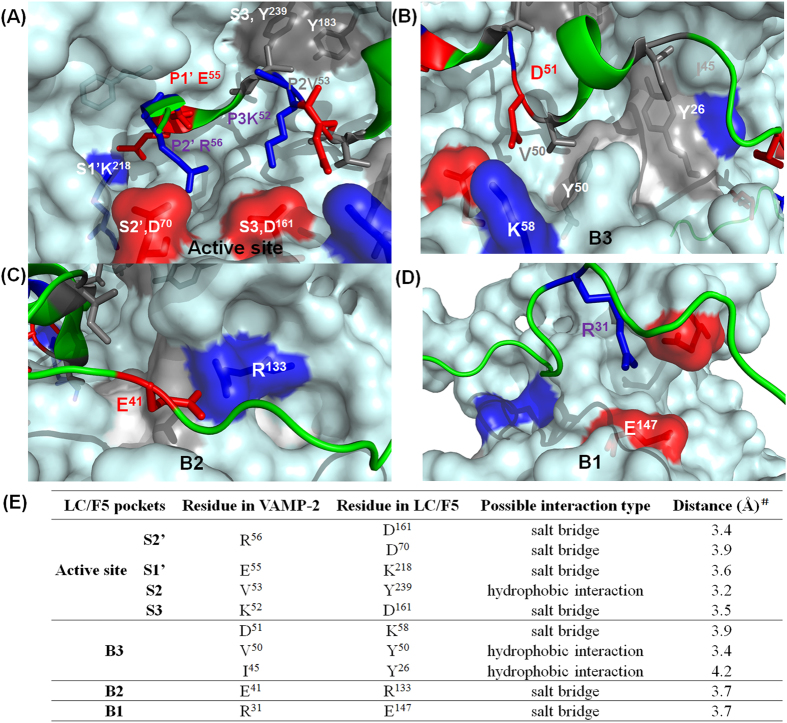
Specific interactions between VAMP-2 and LC/F5. **(A)** At the active site of LC/F5, the S2′ pocket in LC/F5 that specifically recognizes the positively charged residue R^56^ of VAMP-2 comprises two aspartic acids, D^70^ and D^161^. A salt bridge formed between residue D^161^ of LC/F5 and the P2′ residue of VAMP-2 contributes partially to the interaction. The corresponding S1′ pocket that specifically recognizes the P1′ site of VAMP-2 (E^55^) in LC/F5 contains a positively charged residue, K^218^. In the corresponding interaction site of LC/F5, two hydrophobic residues, Y^183^ and Y^239^ interact complementarily and synergistically with the P2 site of VAMP-2 (V^53^). In the S3-P3 interaction, the D^161^ of LC/F5 partially interacts with the K^52^ of VAMP-2 via salt bridge. (**B**) In the B3 pocket of LC/F5, the salt bridge between D^51^ of VAMP-2 and K^58^ of LC/F5, and the hydrophobic or hydrogen bond interactions between I^45^ and V^50^ of VAMP-2 and Y^26^, Y^50^ and T^192^ of LC/F5, are likely to play a role in stabilizing the binding of VAMP-2 with LC/F5 and orienting the scissile bond toward the active site to prompt substrate hydrolysis. **(C)** Like the B3 site/region interactions, another salt bridge interaction between E^41^ of VAMP-2 and R^133^ in the B2 site/region of LC/F5 are likely to exert an effect in stabilizing the interactions of VAMP-2 and LC/F5 and orienting the scissile bond to prompt hydrolysis by LC/F5 as well. (**D**) In the B1 site/region interactions, the residues E^147^ and E^308^ of LC/F5 possibly interact with residue R^31^ of VAMP-2 through salt bridge formation, with the residue E^147^ playing a dominant role. The interactions may initiate the recognition and binding between VAMP-2 and LC/F5. The structure of LC/F5 in the right panel is shown as surface with 40% transparency, the residues of VAMP-2 that were found to interact with the corresponding residues of LC/F5 were shown in stick. The active site recognition and binding site interactions of LC/F5 were shown in stick and highlighted based on side chain: negatively charged (red), positively charged (blue) and hydrophobic (gray); (**E**) The nature and distances of interacting residues.

LC/F5 was found to cleave the VAMP-2 P2′ site mutant, VAMP-2 (R^56^A), with ~4000-fold lower efficiency (Fig. 1), indicating that the VAMP-2 P2′ site played an important role on the hydrolysis of LC/F5. Based on the LC/F5-VAMP-2 complex structure, the S2′ pocket in LC/F5 that specifically recognized the positively charged P2′ site at residue R^56^ of VAMP-2 comprised one negatively charged aspartic acid, D^70^ and D^161^ (Fig. 5A). LC/F5 mutations, D^70^A and D^161^A, caused a ~10-fold and ~1000-fold reduction in the efficiency of VAMP-2 hydrolysis respectively, mainly affecting the substrate catalysis, *k*_cat_ (Table 1). In addition, the double mutations, LC/F5 (D^70^A/D^161^A), were found to completely abolish LC/F5 activity.

#### LC/F5 P1, -S1, substrate recognition

Previous studies proved that the P1′ site of VAMP-2 or SNAP-25 played an important role on recognition and hydrolysis by BoNTs^25^,^26^,^27^,^28^,^29^. In the present work, the substrate P1′ site mutation, VAMP-2 (E^55^A), was found to reduce the LC/F5 hydrolysis efficiency by ~750-fold, indicating a very important role of the VAMP-2 P1′ site in the recognition by LC/F5 (Fig. 1). The S1′ pocket of LC/F5 was predicted to comprise a positively charged residue, K^218^ (Fig. 5A). The LC/F5 (K^218^A) or LC/F5 (K^218^D) mutations could completely abolished the catalytic activity of LC/F5, whereas the LC/F5 (K^218^R) mutation displayed ~8-fold reduction effect on substrate hydrolysis (Table 1). Taken together, the data indicated that the salt bridge between K^218^ of LC/F5 and E^55^ of VAMP-2 played important role on substrate recognition (Fig. 5E).

#### Dual recognition of VAMP-2 P2 (V^53^ )by the S2 pocket of LC/F5

The P2 site of VAMP-2 plays a moderate role in LC/F5 substrate recognition, with the V^53^A change in VAMP-2 reducing LC/F5 substrate hydrolysis by ~10-fold (Fig. 1). Two hydrophobic residues in LC/F5, Y^183^ and Y^239^, were predicted to interact with V^53^ of VAMP-2 (Fig. 5A). The single alanine substitution, Y^183^A or Y^239^A, exerted ~3-fold and ~8-fold reduction on the LC/F5 substrate hydrolysis respectively, but the double mutation of LC/F5, which resulted in the Y^183^A/Y^239^A changes, displayed ~1000-fold reduction on efficiency of substrate hydrolysis, with the effect mainly on *k*_cat_ but not *K*_m_ (Table 1). These data indicated that the hydrophobic pocket formed by Y^183^ and Y^239^ exhibited the best interactive effects with V^53^ of VAMP-2, and that the effect of loss of one Tyrosine site could be compensated by the other (Fig. 5E). This dual recognition strategy has previously been observed in the case of LC/D substrate recognition^26^.

#### LC/F5 P3-S3 substrate recognition

The alanine substitution of the P3 site of VAMP-2, K^52^A, exerted ~20-fold reductive effect on the substrate hydrolysis by LC/F5 (Fig. 1). An aspartic acid, D^161^, in LC/F5 was predicted to partially interact with the K^52^ of VAMP-2 based on the modeled LC/F5-VAMP-2 complex structure (Fig. 5A). The LC/F5 (D^161^A) mutant displayed ~1000-fold attenuated VAMP-2 cleavage efficiency by mainly affecting *k*_cat_ (Table 1). As shown above, the LC/F5 (D^161^A) mutant displayed a significant effect on the substrate hydrolysis efficiency compared to LC/F5(D^70^A) might be due to the fact that residue D^161^ of LC/F5 may exert simultaneous contribution to both of the P2′ and P3 sites of VAMP-2 via salt bridge interactions (Fig. 5A,E).

### LC/F5 binding pockets interactions with VAMP-2

Three binding pockets, designated as B1–B3, in LC/F5 were identified based on the modeled LC/F5-VAMP-2 complex structure (Fig. 4).

#### LC/F5 B3 pocket

The pockets in LC/F5 that specifically recognize the B region/site of VAMP2 were designated as B pockets. The VAMP-2 B3 region, which is located adjacent to the scissile bond, is a VAMP-2 helix with a hydrophobic surface that comprised the residues I^45^, V^50^ and D^51^ (Fig. 5B). The amino acid substitutions of I^45^A, V^50^A and D^51^A in VAMP2 exerted ~4-, ~50- and ~10-fold reduction on LC/F5 substrate hydrolysis respectively (Fig. 1). In the B3 pocket of LC/F5, two tyrosines (Y^26^ and Y^50^) and one polar residue (T^192^) were predicted to interact with the B3 site of VAMP2 in which residue Y^26^ of LC/F5 was likely to interact with I^45^ of VAMP-2 through hydrophobic interaction, and the residues Y^50^ and T^192^ were expected to recognize residue V^50^ of VAMP-2 via hydrophobic or hydrogen bond interactions (Fig. 5B,E). The amino acid changes of LC/F5 (Y^26^A), LC/F5 (Y^50^A) and LC/F5 (T^192^A) reduced substrate hydrolysis efficiency by ~60-, ~10- and ~3-fold respectively. The triple mutations which resulted in the formation of the mutant protein of LC/F5 (Y^26^A/Y^50^A/T^192^A) were found to reduce the hydrolytic activity of LC/F5 on VAMP-2 by ~600-fold (Table 1). In addition, one positively charged residue in the B3 pocket of LC/F5 (K^58^) (Fig. 5B) was predicted to interact with residue D^51^ of VAMP-2 and the K^58^A substitution exerted ~250-fold reduction on LC/F5 substrate hydrolysis (Table 1). Surprisingly, the B3 site mutation exhibited effects mainly on substrate catalysis (*k*_*cat*_), but not substrate binding (*K*_*m*_), suggesting that recognition at the B3 site of VAMP-2 by LC/F5 pocket facilitates the orientation and tuning of substrate for further active site recognition and hydrolysis, in addition to its primary role on substrate binding.

#### LC/F5 B2 pocket

In VAMP-2, the B2 site comprised residue E^41^ (Fig. 5C). The VAMP-2 substitution, E^41^A, exhibited ~80-fold reduction LC/F5 substrate hydrolysis (Fig. 1). The LC/F5 residue, R^133^, was predicted to interact with the residue E^41^ of VAMP-2 via formation of a salt bridge (Fig. 5C,E). The LC/F5 mutation which resulted in the R^133^A change caused ~300-fold reduction on LC/F5 activity (Table 1), indicating the importance of interaction between E^41^ of VAMP-2 and R^133^ of LC/F5. The B2 pocket recognition by LC/F5 mediated by E^41^ of VAMP-2 and R^133^ of LC/F5 may play an important role in stabilizing the binding of VAMP-2 to LC/F5 to facilitate further interaction between VAMP-2 and LC/F5 in the B3 pocket.

#### LC/F5 B1 pocket

The B1 site of VAMP-2, which is distal to the active site, comprised the residue R^31^, which was recognized by the B1 pocket of LC/F5, in which E^147^ and E^308^ were the main residues (Fig. 5D). The VAMP-2 mutation which resulted in the R^31^A substitution reduced LC/F5 hydrolysis by ~200-fold (Fig. 1), suggesting that residue R^31^ was important for LC/F5 substrate recognition. Based on the modeled LC/F5-VAMP-2 complex structure, we conclude that the possible salt bridge formed between R^31^ of VAMP-2 and the residues E^147^ and E^308^ of LC/F5 plays a role in substrate recognition and binding (Fig. 5D,E). Surprisingly, the E^147^A and E^308^A changes in LC/F5 exerted almost no effect on substrate cleavage (data not shown), nor did the double amino acid changes of LC/F5 (E^147^A/E^308^A) (Table 1). The charge reversal substitution of E^147^R, displayed ~80-fold reduction on LC/F5 substrate hydrolysis, whereas the E^308^R change in LC/F5 did not exert an apparent effect on the substrate cleavage activity (Table 1), suggesting that interaction between R^31^ of VAMP-2 and E^147^ of LC/F5 may be important for LC/F5 substrate recognition.

## Discussion

In the present report, the detailed substrate recognition and cleavage mechanism employed by LC/F5 was uncovered for the first time. Saturation mutagenesis analysis on substrate VAMP-2 revealed that the minimal peptide region required for efficient substrate cleavage by LC/F5 was VAMP-2 (20–65), in which both the P1′ (E^55^) and P2′ (R^56^) site residues played a very significant role in substrate recognition by LC/F5 (Fig. 1), which is different from other serotypes of BoNT, for which the P1′ residue played the most important role on substrate hydrolysis^25^,^26^,^28^. The general mechanism of substrate recognition by LC/F5 could be summarized as follows based on our biochemical data. LC/F5 recognition of substrate VAMP-2 was initiated through interaction between the B1 site of VAMP-2 (R^31^) and the B1 pocket of LC/F5, which is composed of the residue E^147^. The R^31^-E^147^ interaction was thought to be important in initiating the first step of substrate recognition. LC/F5 then further interacted with the B2 site of VAMP-2 which comprised E^41^, a key residue recognized by the B2 pocket of LC/F5, which comprised residue R^133^, via the formation of a salt bridge. Interaction between these two binding sites facilitates further binding of LC/F5 to the B3 site of VAMP-2 through multiple interactions including hydrophobic interaction between I^45^ and V^50^ of VAMP-2 and Y^26^, Y^50^ and T^192^ of LC/F5, as well as formation of a salt bridge between D^51^ of VAMP-2 and K^58^ of LC/F5. The B3 pocket recognition event facilitates stabilization and fine tuning of the VAMP-2 structure for more efficient LC/F5 active site recognition rather than strong substrate binding. The initial substrate binding and stabilization between VAMP-2 and LC/F5 facilitate further recognition of the VAMP-2 scissile bond by the active site of LC/F5, where P3-S3, P2-S2, P1′-S1′ and P2′-S2′ interactions and recognitions were mediated by various interaction events including salt bridge interaction between K^52^-D^161^ (P3-S3), the hydrophobic interactions between the V^53^-Y^183^/Y^239^ (P2-S2), and the E^55^-K^218^ (P1′-S1′) and the R^56^-D^161^/D^70^ (P2′-S2′) salt bridges. Interestingly, our results indicate that amino acid D^161^ of LC/F5 may simultaneously interact with residues in the P3 (K^52^) and P2′ (R^56^) sites of VAMP-2 (Fig. 5A). These interactions stabilize the alignment of P1 and P1′ sites of VAMP-2 onto the zinc ion located at the active site of LC/F, and initiate the substrate hydrolysis process.

**Figure 6:**
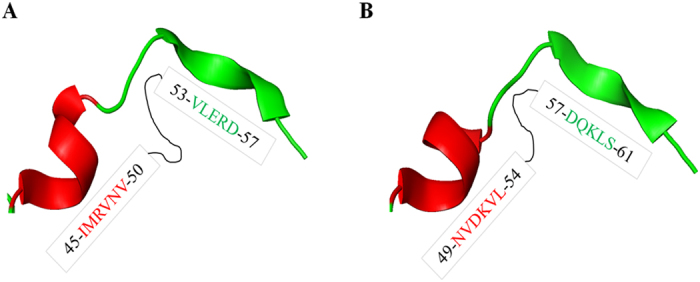
An overall comparison of the four-residue N-terminal shifted α-helix in the VAMP-2 structure. When comparing the modeled LC/F5-VAMP-2 (**A**) and the LC/F1-VAMP-2 (**B**) complex structures, the VAMP-2 α-helix in the later was found to be shifted four residues toward the N-terminal, the shift prompts VAMP-2 to better fit the cleft in the LC/F5 binding pockets and active site for efficient substrate cleavage. The modeled VAMP-2 structure is represented in cartoon with the shifted α-helix colored in red, and so were the corresponding residues. The VAMP-2 structure in the modeled LC/F1-VAMP-2 complex was extracted and refined from reference^25^ with permission.

Results of biochemical characterization of different LC/F5 residues based on the prediction of LC/F5-VMAP-2 complex structure are consistent with our modeled complex structure. Comparison of the mechanisms of substrate recognition employed by LC/F1 and LC/F5 could provide insight into the evolution of substrate recognition by LC/F subtypes. From the modeled and biochemically proven complex structure of LC/F5-VAMP-2 (Fig. 6A), the helix in VAMP-2 was formed by residues I^45^MRVNV^50^, whereas in the complex structure of LC/F1-VAMP-2^25^ (Fig. 6B), it was formed by residues N^49^VDKVL^54^, with one α-helix shift of in the natural structure of VAMP-2. Both helixes provide a hydrophobic interface for interaction with LC/F subtypes. The use of different helix in LC/F1 and LC/F5 is probably due to the different composition in the B1 and B2 recognition pockets, which will determine the initial substrate recognition sites in LC/F1 and LC/F5 respectively, and indirectly the efficiency of priming of the subsequent substrate recognition steps. The data probably suggest the significance of initial substrate binding and recognition. The alternative explanation of the different substrate recognition mechanisms of LC/F1 and LC/F5 is that the active site of BoNT is important for substrate recognition. For LC/F1 and LC/F5, the differential active site composition, in particular the composition of S1′ pocket, determines the efficiency of recognition of different scissile bond. The oppositely charged amino acids in the S1′ pocket, namely K^218^ and E^200^ in LC/F5 and LC/F1 respectively, play a role in determining whether they can recognize the same scissile bond. LC/F5 is expected to scan through VAMP-2 to find the appropriate scissile bond, which will then determine the subsequent substrate binding process. We tend to prefer the former model of evolutional recognition of substrate VAMP-2 due to the commonly accepted concept that BoNT substrate recognition was initiated from the distal binding site.

To conclude, this study uncovered the step by step recognition of VAMP-2 by LC/F5 and provided direct evidence of the evolution of novel botulinum neurotoxin through changing its cleavage site on the known substrate while maintaining the overall similar substrate binding and recognition mode.

## Experimental Materials and Methods

### LC/F expression and purification

To facilitate the expression of target protein, the corresponding sequence to full length LC/F5 residues 1–450 (GenBank: ADA79579.1) was optimized for the codons preferred in *E. coli*, then synthesized by Tech Dragon (Hong Kong, China) and sub-cloned into a pGEX-2T vector through the *SacI/BamHI* restriction sites. In addition, the human VAMP-2 (1–97) construct was synthesized as described previously^28^. All the mutated derivatives of LC/F5 and VAMP-2 were performed by using the QuickChange (Stratagene) commercial kit following the manufacturer’s instruction and confirmed by sequencing in BGI (Shenzhen, China). Based on our previous study of the expression and characterization of LC/F5^24^, GST tagged full length LC/F5 (1–450) were found to display the highest solubility and activity when compared with other versions of LC/F5. Thus in the present study, all the assays were carried out by using purified GST-LC/F5 (1–450) protein. Purification of GST-LC/F5 (1–450), VAMP-2 (1–97) and all other derivatives was performed as previously described^24^,^30^.

### Standard Linear Velocity Reaction

Linear velocity assays were performed as previously described^30^. Briefly, in 10 μl reaction mixture, 5–10 μM VAMP-2 or derivatives was mixed with an indicated amount of LC/F5 in 10:20 buffer (10 mM Tris-HCl, pH 7.6, 20 mM NaCl). After 20min incubation at 37 °C, the reactions were stopped by adding SDS-PAGE sample buffer, heated at 100 °C for 5min, analyzed by SDS-PAGE. The amount of VAMP-2 cleaved was determined by densitometry.

### Determination of Kinetic Parameters

As described previously^26^, the procedures for *K*_*m*_ and *k*_*cat*_ determination were almost the same as mentioned above, but the amount of LC/F5 or its derivatives used was adjusted to achieve <10% cleavage of VAMP-2, the concentrations of which ranged from 1 to 72 μM. Reaction velocity against substrate concentration was fitted into the Michaelis–Menten equation and kinetic constants were derived using the GraphPad program. For each protein, at least three independent assays were performed to determine the kinetic constants.

### Far-UV Circular Dichroism Analysis

As detailed previously^26^, LC/F5 and derivatives were analyzed by far-UV CD for assessing the secondary structure change in a 10-mm path length quartz cuvette of 400 μl volume (containing 0.1–0.4 mg/ml protein in 10:20 buffer). A JASCO J-810 spectropolarimeter was used with the following parameters: scanning speed 50 nm/min, 1s response time, 1nm data pitch, 1nm band width and accumulation times was set as 3. The wavelength range of 190–250nm was scanned, and raw CD data were converted to molar ellipticity using Yang as reference^31^, and the spectrum was generated using GraphPad Prism.

### Molecular Modeling

The complex structure of LC/F5-VAMP-2 was modeled by using SWISS-MODEL and refined with PyMoL software as detailed previously^32^. Briefly, the structure of LC/F5 (1–450) was modeled by SWISS-MODEL using the crystal structure of LC/F (PDB 2A8A) as searching template and the structure of VAMP-2 was extracted from the SNARE complex crystal structure (chain A, PDB 1SFC), and both structures were modified by PyMoL. The LC/F5-VAMP-2 complex structure was then modeled by aligning to the LC/F-VAMP-2 inhibitor complex structure (PDB 3FIE) and refined in the PyMoL software.

## Additional Information

**How to cite this article**: Guo, J. *et al.* Mechanism of substrate recognition by the novel Botulinum Neurotoxin subtype F5. *Sci. Rep.* **6**, 19875; doi: 10.1038/srep19875 (2016).

## Figures and Tables

**Table 1:**
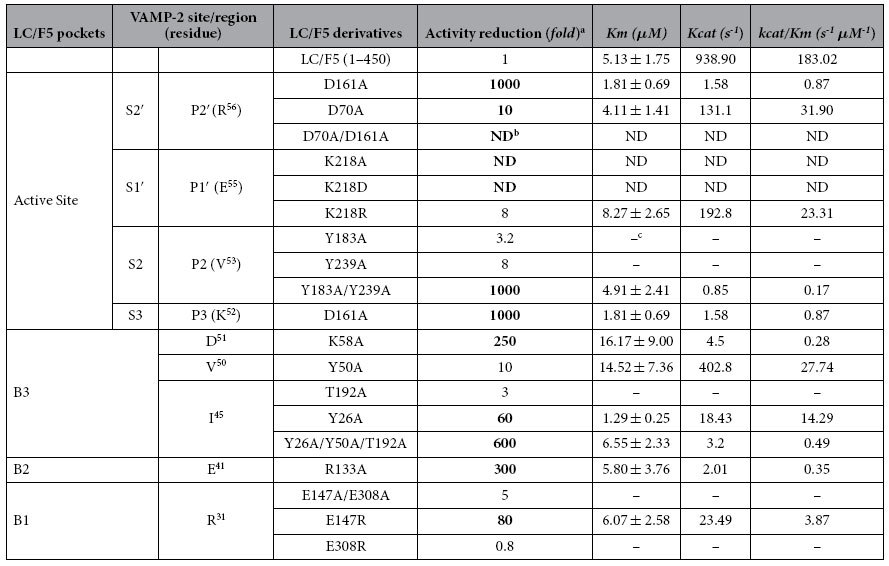
Efficiency of VAMP-2 hydrolysis and kinetic constants of LC/F5 and derivatives.

^a^The ratio of the amount of LC/F5 derivatives/LC/F5 wtneeded to cleave 50% of VAMP-2 wt.

^b^ND, not detectable. The mutant was too inactive for determination ofits kinetic constants in the present study.

^c^kinetic constants were not determined.
